# Denoising of ASL Data Using Deep Learning Priors Generated From Distribution Remapping

**DOI:** 10.1002/mrm.70471

**Published:** 2026-06-10

**Authors:** Ziyang Xu, Rong Guo, Ziwen Ke, Yudu Li, Yibo Zhao, Wen Jin, Ruihao Liu, Ziyu Meng, Yao Li, Zhi‐Pei Liang

**Affiliations:** ^1^ Beckman Institute for Advanced Science and Technology, University of Illinois at Urbana‐Champaign Urbana Illinois USA; ^2^ Department of Electrical and Computer Engineering University of Illinois at Urbana‐Champaign Urbana Illinois USA; ^3^ Siemens Medical Solutions USA, Inc. Malvern Pennsylvania USA; ^4^ National Engineering Research Center of Advanced Magnetic Resonance Technologies for Diagnosis and Therapy, School of Biomedical Engineering, Shanghai Jiao Tong University Shanghai China; ^5^ National Center for Supercomputing Applications, University of Illinois at Urbana‐Champaign Urbana Illinois USA; ^6^ Department of Bioengineering University of Illinois at Urbana‐Champaign Urbana Illinois USA

**Keywords:** arterial spin labeling, deep learning, denoising, distribution remapping

## Abstract

**Purpose:**

To develop an effective deep learning (DL)–based method to denoise arterial spin labeling (ASL) data.

**Methods:**

Conventional DL–based ASL denoising methods often suffer from overfitting and poor generalization when training data are limited. The proposed method overcame this problem using two strategies: (i) perform data augmentation to create large training data and (ii) denoise in‐distribution and out‐of‐distribution components of the target perfusion‐weighted image separately. Specifically, Image‐to‐Image Schrödinger Bridge (I^2^SB)–based distribution remapping transforms were applied to the large public ASL datasets so that their intensity distribution matched that of the data to be denoised. U‐Net–based DL denoisers were trained on the remapped data to capture in‐distribution features. High‐SNR outputs from the DL‐denoiser were incorporated into a Bayesian model to reconstruct the out‐of‐distribution features with sparsity constraints, generating denoised images for cerebral blood flow (CBF) quantification.

**Results:**

Simulation studies highlighted the importance of distribution remapping for effective data augmentation in limited‐data scenarios. Both simulation and in vivo experiments showed that the proposed method outperformed state‐of‐the‐art approaches, achieving an average SNR improvement of approximately 7 dB. Evaluations on multiple datasets confirmed robust and generalizable performance across different ASL sequences and imaging protocols. To demonstrate clinical potential, our method was applied to denoising stroke patient data (using only one‐sixth of total averages with ˜83% reduction in scan time) and produced comparable CBF maps to the conventional ASL method.

**Conclusion:**

The proposed method enables effective ASL denoising with limited training data. It has the potential to accelerate ASL acquisition, enhance image quality, and improve clinical utility.

## 1 Introduction

Arterial spin labeling (ASL) measures the cerebral blood flow (CBF) by magnetically labeling arterial blood water as an endogenous tracer [1], which has the potential to characterize neurovascular diseases [2, 3], dementia [4, 5] and brain tumors [6, 7]. However, ASL data often suffer from low signal‐to‐noise ratio (SNR), leading to increased variability and reduced reliability of CBF measurements [8, 9]. To gain sufficient SNR, multiple signal averages are typically required, which substantially increases the scan time [8, 10, 11]. As a result, the practical utility of ASL is still limited despite its clinical and research potential.

Over the past decades, substantial efforts have been made to improve the SNR of ASL data. In data acquisition, several advanced methods, such as pseudo‐continuous ASL (pCASL) [12] and 3D ASL (e.g., GRASE [13] or spiral readouts [14]), have been developed, which significantly improve ASL image quality. In data processing, various methods have been developed. Conventional model–based methods exploit intrinsic signal characteristics, such as spatial smoothness [15, 16], sparsity in the wavelet domain [17, 18], non‐local similarity [19, 20], and low‐rankness [21, 22, 23, 24]. These methods often rely on strong spatial regularization or handcrafted image features, which may cause significant bias and thus limit performance. More recently, deep learning (DL)–based methods using convolutional neural networks (CNNs) [25, 26, 27, 28, 29, 30], transformer‐based networks [31, 32, 33], variational autoencoder (VAE) [34], generative adversarial network (GAN) [35], and diffusion models [36], have shown great potential for ASL denoising by utilizing extrinsic priors learned from training data. However, while several large‐scale ASL/CBF datasets are publicly available [37, 38, 39], their image characteristics often vary substantially due to the use of different labeling schemes (CASL [40, 41], PASL [42, 43], or pCASL [12, 44]), readout trajectories (EPI [10], GRASE [13, 44, 45], or TSE [46, 47]) and sequence parameters (spatial resolution, echo time, post labeling delay, etc.). As a result, the training data available for a specific ASL sequence is still very limited, leading to poor generalization of the DL models. Another major problem of existing DL‐based methods is the potential bias toward training data, resulting in the loss of novel image features outside of the training distribution, such as pathological structures in patient data.

In this paper, we propose a new method to overcome the problems with DL‐based denoising of ASL data. The proposed method uses a distribution‐remapping strategy to address the issue of limited training data and uses sparse modeling to recover the novel features outside the training distribution. Evaluated using both simulated and experimental data, the proposed method showed robust and generalizable denoising performance across diverse low‐SNR ASL datasets. This method may enhance the practical utility of ASL in research and clinical applications.

## 2 Theory

### 2.1 Image Model

In single post‐label‐delay ASL acquisitions, perfusion‐weighted image (denoted as ΔM) is typically measured as the label‐control differences, and a separate reference image (denoted as $M_{0}$) is acquired for CBF (denoted as f) quantification. The measured perfusion‐weighted image $\Delta M_{n}$ can be expressed as:

(1) $$\Delta M_{n}=\Delta M_{t}+\xi ,$$

where $\Delta M_{t}$ represents the desired noise‐free perfusion‐weighted images, and ξ denotes the measurement noise, which follows a Rician distribution. Separating the desired signals from noise is very challenging for ASL data with low SNR, which is often the case for accelerated ASL acquisitions with a smaller number of averages of the conventional acquisitions.

The proposed method decomposes the desired perfusion‐weighted image into two components:

(2) $$\begin{array}{c} \begin{array}{c} \Delta M_{t}=\Delta M_{r}+\Delta M_{s},\\ \text{subj}.\;\text{to}\;\Delta M_{r}=A_{\beta }^{-1}\left(f_{r}\right)\;\text{and}\;{\left\|\nabla \left(\Delta M_{s}\right)\right\|}_{1}\leq \delta , \end{array} \end{array}$$

where $\Delta M_{r}$ represents a reference component representing “in‐distribution” normal image features learned and predicted using DL, and $\Delta M_{s}$ is a sparse residual component capturing “out‐of‐distribution” subject‐specific novel features beyond the reference prior. ∇ is the total variation (TV) operator. The reference perfusion‐weighted image $\Delta M_{r}$ is generated from a reference CBF map ($f_{r}$) through the inverse Buxton model $A_{\beta }^{-1}\left(\cdot \right)$.

In this model, the reference component and sparse component serve complementary roles [48, 49] (see Figure S1 for an illustration). The reference component is designed to capture normal features that are predictable from population data, which provides a high‐SNR, high‐quality prior reflecting the population‐level statistics. More specifically, the “in‐distribution” image features are represented as a denoising network trained on the populational prior data. It is represented in the form of reference CBF map $f_{r}$ due to the quantitative nature and the wider availability of CBF population data. The sparse component is designed to account for the subject‐specific deviations, including pathological features such as tumors or ischemic lesions not presented in training data from healthy subjects. A transformed sparsity constraint is imposed on this component for effective reconstruction, since the novel features (beyond the normative part) are typically localized and sparse. Together, this model enables the use of stronger priors for the reference component and effective recovery of sparse features that are out of the training distributions.

**FIGURE 1:**
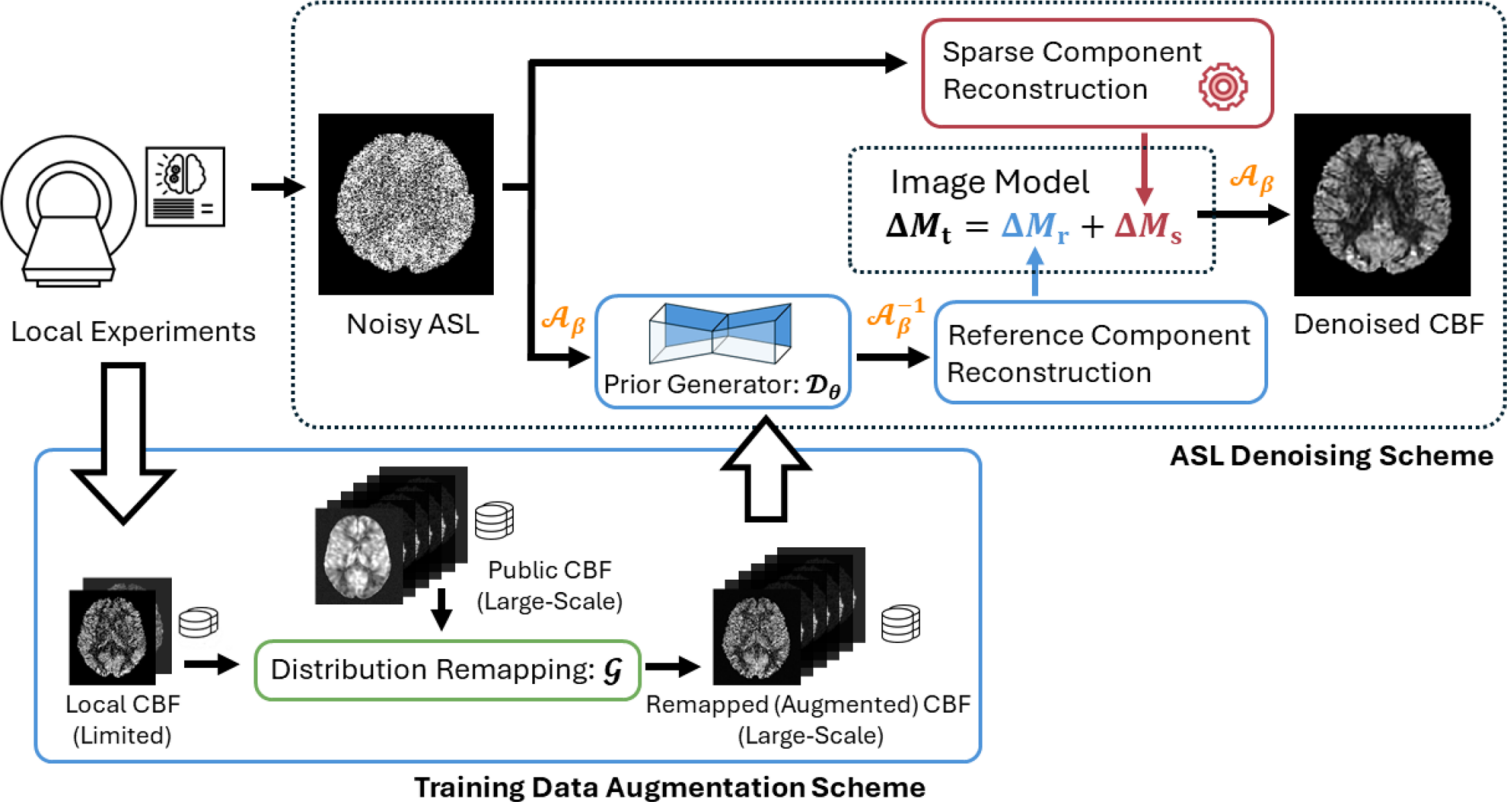
Illustration of the proposed denoising method. Our method decomposes the desired perfusion‐weighted image into two components: the reference component ($\Delta M_{r}$) and the sparse component ($\Delta M_{s}$). The reference component is designed to capture normal features that are predictable from population data. This component is predicted by a denoising neural network trained on public CBF data whose distribution is remapped to the one of the target data. The sparse component is designed to account for the subject‐specific deviations, including pathological features such as tumors or ischemic lesions not present in training data from healthy subjects.

As illustrated in Figure 1, the reference CBF $f_{r}$ was constructed using a denoising neural network trained on high‐ and low‐SNR data pairs that were generated from large public data (as described in Section 2.2). Then $f_{r}$ was used to generate the reference component $\Delta M_{r}$. The sparse component was recovered by a sparsity‐constrained Bayesian model. The following sections describe these steps in detail.

### 2.2 Distribution Remapping–Based Deep Prior Generation

We used a DL model to learn the nonlinear mapping from noisy CBF estimates ($f_{n}$) to their high‐SNR counterparts ($f_{r}^{*}$):

(3) $$\begin{array}{c} f_{r}^{*}=D_{\theta }\left(f_{n}\right). \end{array}$$

Here, $f_{n}$ is obtained from the measured perfusion‐weighted image $\Delta M_{n}$ via the Buxton model, that is, $f_{n}=A_{\beta }\left(\Delta M_{n}\right)$. $D_{\theta }$ represents a neural network parameterized by θ, which serves as a CBF denoiser. This model implicitly encodes the statistical priors of CBF from the training data. Therefore, when trained with sufficient data, it is supposed to provide a subject‐matched, data‐consistent CBF reference, capturing image features that reside within the statistical distribution of the training data. In this work, the noisy and high‐SNR reference CBF maps were generated using one‐sixth and the full averages available, respectively. The denoiser was implemented as a U‐Net due to its fast inference. Detailed network architecture is shown in Figure S2.

Acquiring sufficient paired low‐/high‐SNR CBF data for a given ASL sequence used for a specific application is usually impractical. However, large‐scale ASL/CBF datasets such as ADNI [39], QTAB [37], and PTBP [38] are available, which offer broad demographic and physiological diversity. A practical issue in using these public ASL/CBF datasets for training $D_{\theta }$ is that the signal properties of these data often differ substantially from those acquired for a specific application, as demonstrated in Figure S3. To address this issue, we used a distribution remapping strategy based on Diffusion Schrödinger Bridges (DSB) [50, 51, 52] to transform the public data to match the local training dataset. DSB is a generative model based on stochastic optimal transport, to perform domain‐to‐domain translation between the public and local data. This allows synthesis of training data that contain both sequence‐matched signal characteristics and sufficient population variations.

Specifically, to enrich the local high‐SNR CBF dataset, one variant of the DSB model, known as Image‐to‐Image Schrödinger Bridge (I^2^SB) [50], was trained to translate the intensity distribution of public high‐SNR CBF maps to match that of the local ASL data:

(4) $$\begin{array}{c} f_{\mathrm{tar}}=G_{f}\left(f_{\mathrm{pub}}\right), \end{array}$$

where $f_{\mathrm{pub}}$ and $f_{\mathrm{tar}}$ denote the CBF maps following public and local CBF distributions, respectively. Another I^2^SB model was trained to translate Gaussian noise ($\varepsilon_{\text{Gauss}}$) into the noise distribution in the local CBF data ($\varepsilon_{\mathrm{tar}}$):

(5) $$\begin{array}{c} \varepsilon_{\mathrm{tar}}=G_{n}\left(\varepsilon_{\text{Gauss}}\right). \end{array}$$

The model $G_{f}$ was trained using pseudo‐paired data constructed between public and local CBF datasets. Specifically, each public CBF map was nonlinearly registered to each local subject using a diffeomorphic deformation model (ANTs were used for this task [53]), generating structurally aligned pairs with differing intensity and noise characteristics. Although minor residual mismatches might remain, their impact was mitigated by pairing each public image with multiple local subjects, which created an implicit averaging effect during training and encouraged learning of distribution remapping rather than structural transformation. Using these pseudo‐paired data, $G_{f}$ was trained in a standard image‐to‐image translation manner. In parallel, the model $G_{n}$ was trained using pairings between Gaussian noise samples and locally measured residual noise (obtained by subtracting high‐SNR CBF from low‐SNR CBF). Details of how these two distribution‐remapping models and the denoiser model were trained are provided in the Supporting Information.

With the remapped high‐SNR CBF and noise, low‐SNR CBF maps were synthesized by adding them together ($f_{\text{noisy}}=f_{\mathrm{tar}}+\varepsilon_{\mathrm{tar}}$). The resulting pairs $\left(f_{\text{noisy}},f_{\mathrm{tar}}\right)$ served as the desired training samples. As is shown in Figures S4 and S5, this distribution‐remapping strategy effectively aligns the intensity and noise characteristics of public and local CBF data, substantially expanding the size and diversity of local training data by approximately a factor of 100 (we expanded the training data from 7 subjects to 750). The resulting datasets were used to train the network models described in Equation (3), for generating high‐SNR CBF reference from low‐SNR CBF inputs.

### 2.3 Recovery of Sparse Novel Features

With the high‐SNR CBF map generated from the trained network, the reference perfusion‐weighted image was derived through the inverse Buxton model ($\Delta M_{r}^{*}=A_{\beta }^{-1}\left(f_{r}^{*}\right)$). To determine the sparse component $\Delta M_{s}$, which captures the subject‐specific features beyond the populational prior, we used a sparsity‐constrained Bayesian model for reconstruction. Following Equations (1) and (2), the sparse component ($\Delta M_{s}^{*}$) was estimated using the following maximum a posteriori (MAP) formulation:

(6) $$\begin{array}{cc} \Delta M_{s}^{*} & =\arg\max_{\Delta M_{s}}P\left(\Delta M_{s},\Delta M_{n}|\Delta M_{r}^{*}\right)\\ & \;\propto \arg\max_{\Delta M_{s}}\left(\log P\left(\Delta M_{n}|\Delta M_{s},\Delta M_{r}^{*}\right)+\log P\left(\Delta M_{s}|\Delta M_{r}^{*}\right)\right). \end{array}$$

The likelihood term $P\left(\Delta M_{n}|\Delta M_{s},\Delta M_{r}^{*}\right)$ explicitly reflects the characteristics of Rician noise in ASL measurements:

(7) $$\begin{array}{c} \begin{array}{c} P\left(\Delta M_{n}|\Delta M_{s},\Delta M_{r}^{*}\right)=\frac{\Delta M_{t}}{\sigma^{2}}\exp\left(-\frac{{\Delta M}_{n}^{2}+\Delta M_{t}^{2}}{2\sigma^{2}}\right)I_{0}\left(\frac{\Delta M_{n}\cdot \Delta M_{t}}{\sigma^{2}}\right)\\ \text{subj}.\;\text{to}\;\Delta M_{t}=\Delta M_{r}^{*}+\Delta M_{s} \end{array}, \end{array}$$

where $I_{0}\left(\cdot \right)$ denotes the zeroth‐order‐modified Bessel function, and $\sigma^{2}$ is a parameter of Rician distribution that corresponds to the variance of the underlying complex Gaussian noise.

The prior term $P\left(\Delta M_{s}|\Delta M_{r}^{*}\right)$ is designed as a conditional Markov random field that enforces sparsity and structural coherence of the sparse component:

(8) $$\begin{array}{c} P\left(\Delta M_{s}|\Delta M_{r}^{*}\right)\propto \exp\left\{-\lambda_{\mathrm{reg}}{\left\|\nabla \left(\Delta M_{s}\right)\right\|}_{1}\right\}, \end{array}$$

where $\lambda_{\mathrm{reg}}$ represents the regularization parameter controlling the sparsity level. This conditional prior formulation ensures that the reconstructed ASL images maintain edge and anatomical consistency with the high‐SNR reference while allowing sparse deviations that capture subject‐specific novel features. Consequently, the model achieves robust noise suppression without sacrificing the anatomical fidelity provided by the learned priors $f_{r}^{*}$.

Combining the likelihood and prior terms yields the following optimization problem:

(9) $$\begin{array}{cc} \Delta M_{s}^{*} & =\arg\min_{\Delta M_{s}}\frac{\Delta M_{n}^{2}+{\left(\Delta M_{r}^{*}+\Delta M_{s}\right)}^{2}}{2\sigma^{2}}\\ & \;-\log I_{0}\left(\frac{\Delta M_{n}\cdot \left(\Delta M_{r}^{*}+\Delta M_{s}\right)}{\sigma^{2}}\right)+\lambda_{\mathrm{reg}}\;{\left\|\nabla \left(\Delta M_{s}\right)\right\|}_{1}. \end{array}$$

The optimization problem was solved using a quasi‐Newton (BFGS) algorithm [54], which iteratively updates $\Delta M_{s}$ based on the gradient of the objective function. The noise variance $\sigma^{2}$ was estimated from the background regions. The regularization weight $\lambda_{\mathrm{reg}}$ was selected on a per‐subject basis, with the optimal value determined using the L‐curve method [55]. Upon convergence, the denoised perfusion‐weighted image was synthesized as $\Delta M_{t}^{*}=A_{\beta }^{-1}\left(f_{r}^{*}\right)+\Delta M_{s}^{*}$, and the CBF was then derived.

## 3 Methods

### 3.1 Buxton Model for CBF Calculation

Given a specific ASL acquisition protocol, the CBF value in each voxel can be quantified as [56, 57]:

(10) $$\begin{array}{c} f=A_{\beta }\left(\Delta M\right)=\left\{\begin{array}{cc} \frac{6000\cdot \lambda }{2\cdot \lambda \cdot T_{1,\text{blood}}\cdot \left(1-e^{\frac{\tau }{T_{1,\text{blood}}}}\right)}\cdot e^{\frac{\mathrm{PLD}}{T_{1,\text{blood}}}}\cdot \frac{\Delta M}{M_{0}}, & \;\text{for pCASL}\\ \frac{6000\cdot \lambda }{2\cdot \alpha \cdot TI_{1}}\cdot e^{\frac{\mathrm{TI}}{T_{1,\text{blood}}}}\cdot \frac{\Delta M}{M_{0}}, & \text{for PASL} \end{array}\right.. \end{array}$$

where $A_{\beta }$ includes an exponential scale term determined by the sequence‐related parameters $\beta =\left\{\lambda ,\alpha ,T_{1,\text{blood}},\mathrm{PLD},\tau ,TI_{1},\mathrm{TI}\right\}$ and a voxel‐wise normalization term determined by the separately acquired proton density reference $M_{0}$. λ is the tissue/blood partition coefficient (usually 0.9 mL/g), α the labeling efficiency (0.85 for pCASL and 0.98 for PASL), $T_{1,\text{blood}}$ the longitudinal relaxation time of blood (usually 1650 ms for 3T); PLD is the post‐labeling delay, τ the label duration in pCASL, $TI_{1}$ the bolus duration in PASL, and TI the inversion time between the labeling pulse and image acquisition in PASL.

### 3.2 Experimental Data Setup

The proposed method was evaluated on three ASL sequences: (i) a specialized PASL‐3D‐EPSI sequence [58, 59], which was used to acquire ASL data from 10 healthy volunteers on a 3T MR system (MAGNETOM Prisma, Siemens Healthineers, Erlangen, Germany); (ii) a pCASL‐3D‐GRASE sequence [44, 60], which was used to collect data from 10 subjects; and (iii) a PASL‐2D‐EPI sequence, which was used in a subset of the ADNI3 dataset; but we only included data from 18 subjects to simulate limited data condition. All the local datasets were partitioned into training and testing datasets as detailed in Table S1. Two large public ASL datasets, QTAB and PTBP [37, 38, 61], comprising a total of 750 subjects acquired using the pCASL‐2D‐EPI sequence, were employed as the public training data. Two stroke patient datasets acquired using the pCASL‐3D‐GRASE sequence were included to evaluate the feasibility of the proposed method to handle clinical data with lesions that were not present in the training data. CBF maps were quantified from the perfusion‐weighted images and their corresponding proton‐density reference images using the Buxton model described above. For each dataset, high‐SNR CBF maps were generated by averaging all available control/label image pairs, whereas low‐SNR CBF maps were obtained using only 16.7%–20% of the total pairs. Details of all CBF datasets and the usage are summarized in Table S1.

### 3.3 Performance Evaluations

In this study, several existing denoising methods, including denoising with TV [62], statistical constraint–based denoising [55, 63, 64], and deep‐learning–based denoising, were implemented for comparison. Multiple quantitative metrics, including relative mean square error (RMSE), peak signal‐to‐noise ratio (PSNR), and structural similarity index measure (SSIM), were used to assess the denoising performance of the methods. The detailed formulations of these methods and the evaluation metrics are provided in the Supporting Information.

## 4 Results

**FIGURE 2:**
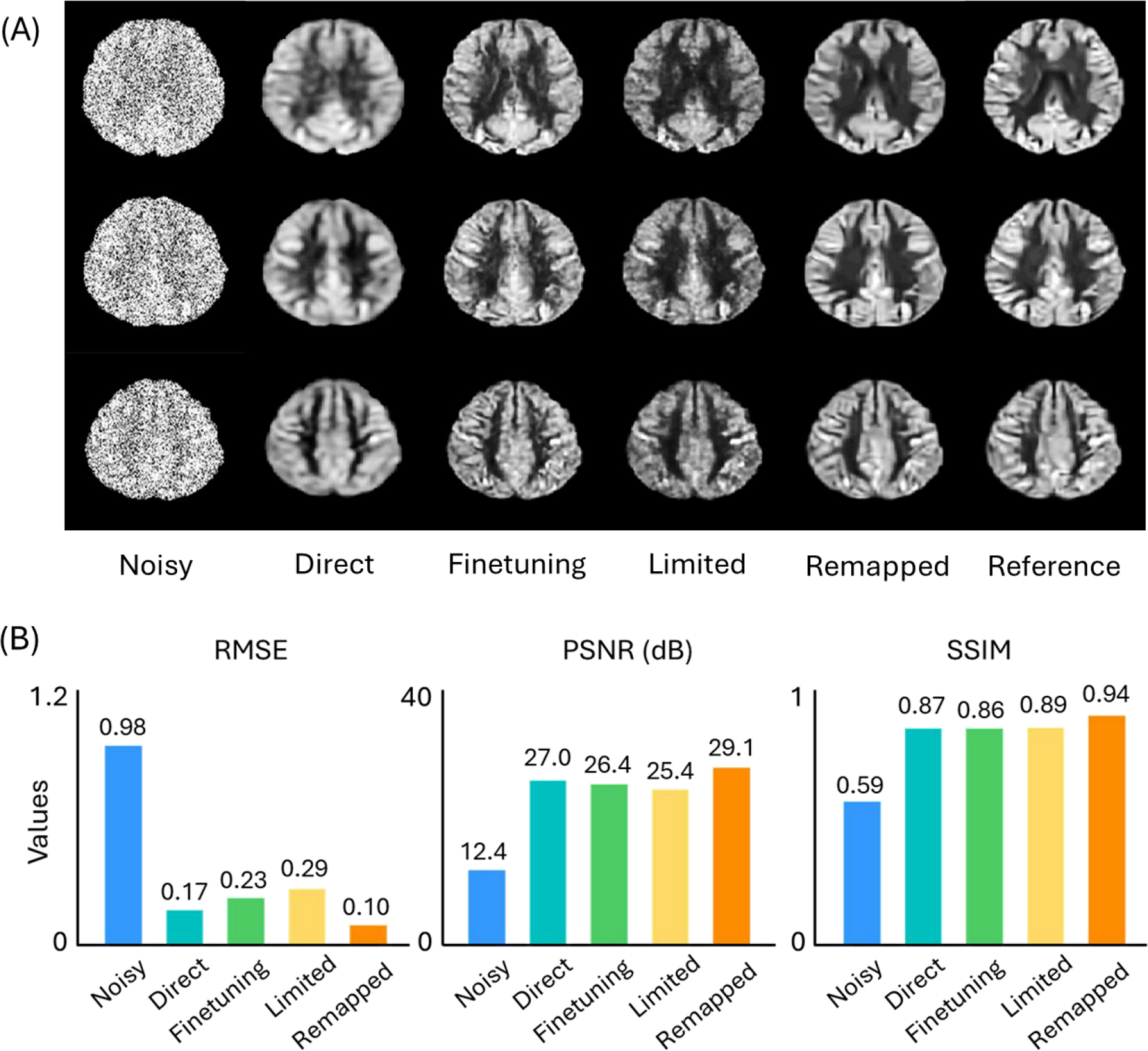
Simulation study demonstrates the role of distribution remapping–based data augmentation in denoising prior learning. (A) Denoising results under different training scenarios. Noisy: noisy measured data (acquired using PASL‐3D‐EPSI sequence); Direct: denoised measured data directly using the pre‐trained denoiser on large public datasets; Finetuning: denoised measured data using a fine‐tuned denoiser with the small‐size measured training data; Limited: denoised measured data using a denoiser trained on the small‐size measured training data; Remapped: denoised measured data using a denoiser trained on the large remapped training data from the proposed data augmentation strategy; Reference: corresponding high‐SNR CBF maps. (B) Quantitative analysis of denoising results under different training scenarios within gray matter (GM).

We first validated the role of the proposed distribution remapping in improving denoising prior learning via simulation. One high‐SNR CBF map from local data was first selected and then added with Rician noise to create its low‐SNR counterpart. The denoising networks with the same architecture were trained under four scenarios and compared: (i) directly on public data; (ii) fine‐tuned on limited local data given (i) [32, 65]; (iii) only on limited local data; and (iv) on augmented local data using the proposed distribution remapping strategy. Figure 2A summarizes the representative denoised results using networks trained under different scenarios. Among the commonly used strategies for handling limited training data, the proposed data augmentation strategy (iv) produced the CBF map that aligned best with reference, preserving both intensity distribution and spatial integrity. Figure 2B summarizes the quantitative comparison across different training scenarios. More ablation studies were conducted to compare different data augmentation methods, as detailed in Figure S6. Denoising with the proposed distribution remapping–based data augmentation achieved the most accurate results.

**FIGURE 3:**
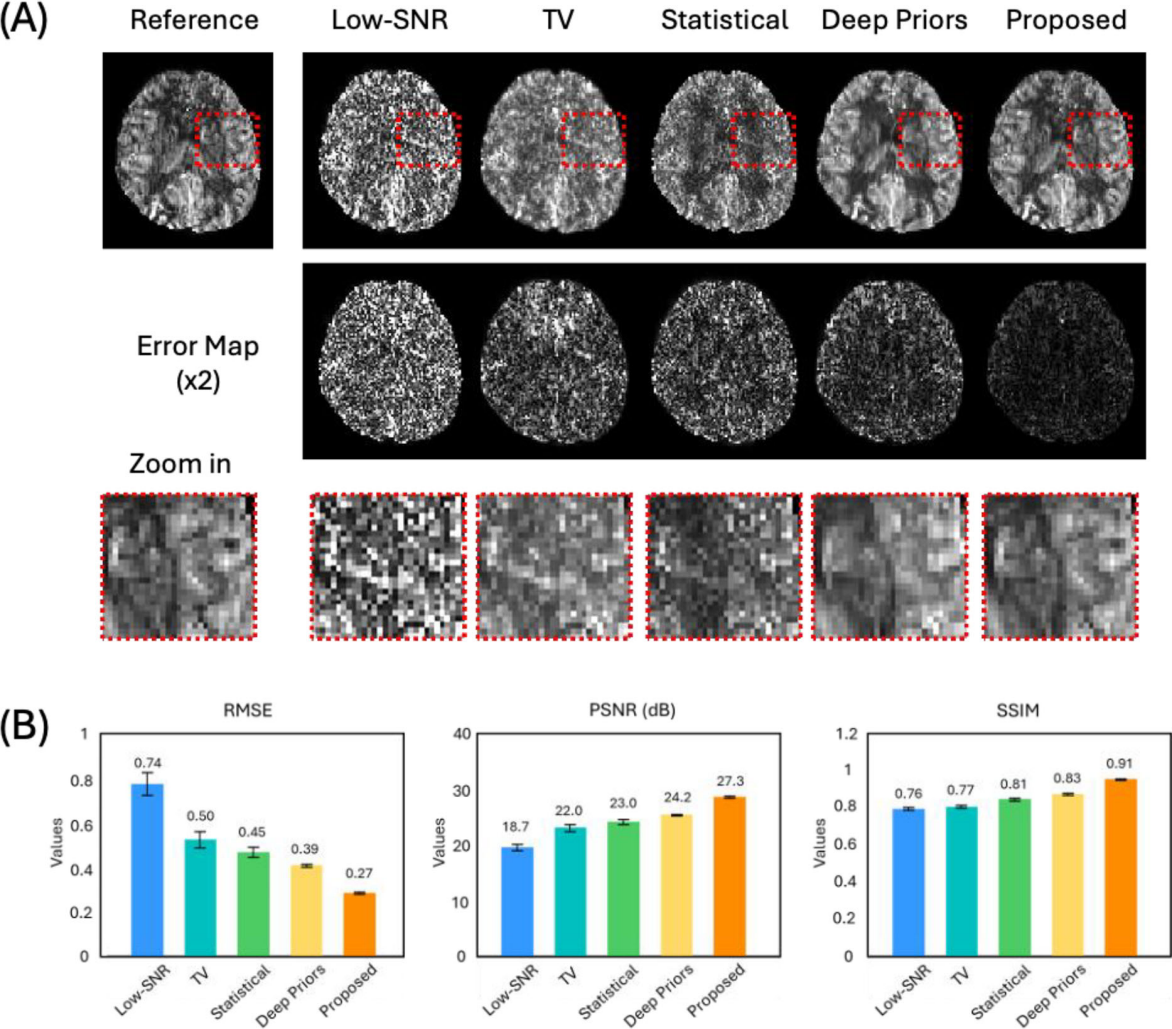
Comparison of denoising methods on in vivo CBF maps. (A) Denoised CBF maps obtained using different denoising methods, where low‐SNR maps are acquired using PASL‐3D‐EPSI sequence with 5 averages (scan time: 42 s) and the high‐SNR reference maps are acquired with 30 averages (scan time: 250 s). (B) Quantitative evaluation of different denoising methods using RMSE, PSNR, and SSIM metrics, collected from five repeated measurements. The proposed method achieved the best noise suppression and the highest structural fidelity relative to the high‐SNR reference. TV, total variation.

Figure 3 compares the proposed method with several existing methods in denoising in vivo ASL images. As shown in Figure 3A, denoising with TV regularization reduced noise but over‐smoothed subtle image details. The statistical constraint–based approach provided moderate denoising but left residual noise and introduced local artifacts. Deep prior images from the trained denoiser achieved stronger noise suppression but showed minor feature loss. The proposed method achieved excellent noise reduction while preserving the cortical and subcortical details. Quantitative comparison across methods is presented in Figure 3B. The proposed approach achieved the lowest RMSE, the highest PSNR, and SSIM.

We also assessed the reproducibility of these methods compared to existing methods. Two low‐SNR CBF maps were generated from separate repetitions then denoised. As shown in Figure S7, the proposed method achieved the smallest mean bias and the narrowest standard deviations bound among all tested methods, indicating highest consistency and reproducibility. More results from different noisy repetitions were shown in Figure S8.

**FIGURE 4:**
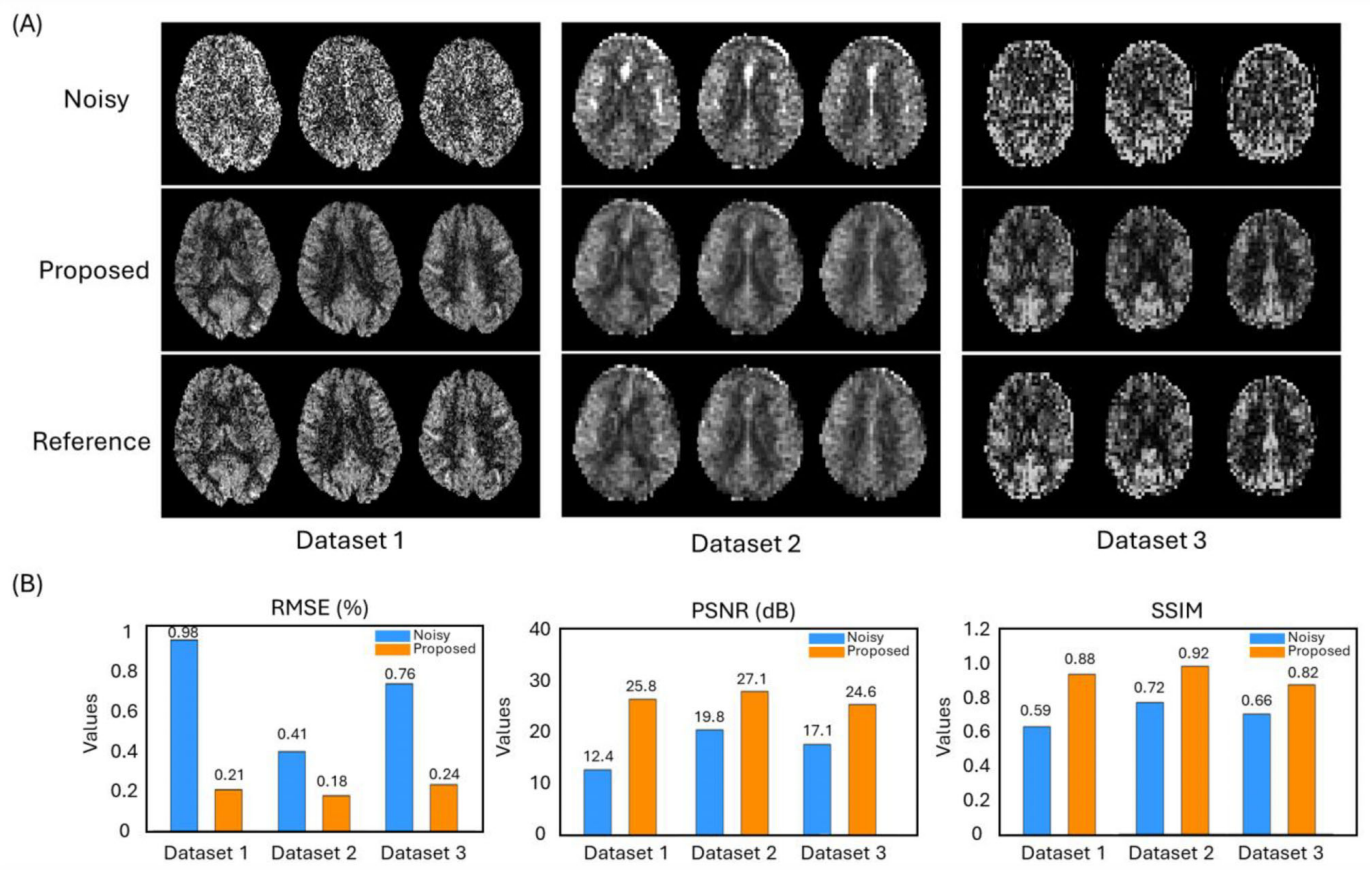
In vivo denoising results on three local datasets with different ASL sequences and readout strategies. (A) CBF images from three local datasets before and after the denoising process. Reference images were generated using full averaging to provide high‐SNR ground truth for comparison. (B) Quantitative analysis of denoising results under different acquisition protocols. Quantitative image quality metrics improved substantially after denoising.

The generalized capability of the proposed data augmentation and denoising method was further validated on three ASL datasets acquired with different sequences. As shown in Figure 4, the denoised CBF maps were consistent with the corresponding reference images across all datasets, demonstrating that the proposed method generalized well to different sequences. More results can be found in Figures S5 and S9.

**FIGURE 5:**
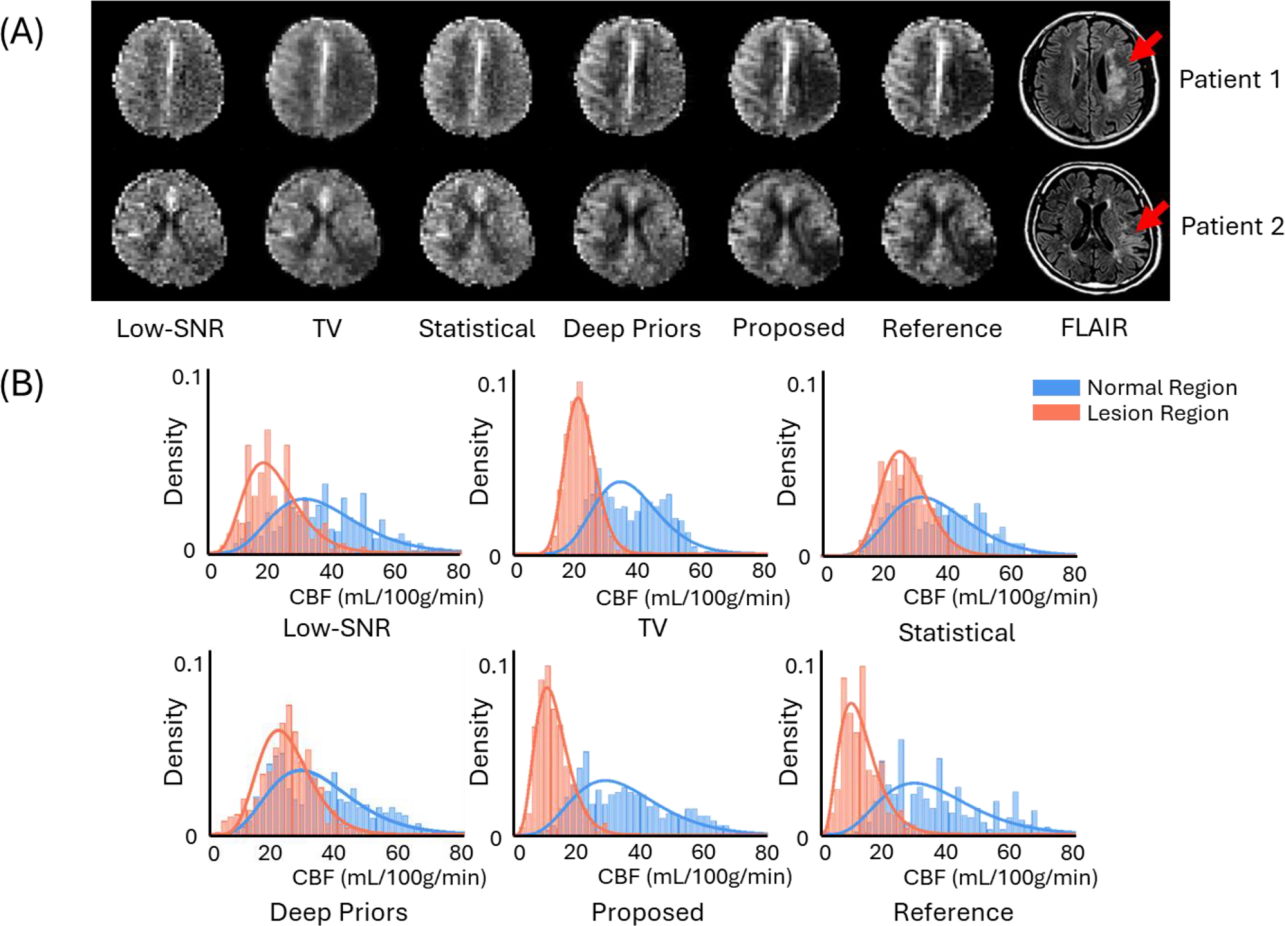
Representative denoised results from two stroke patients. (A) The denoised CBF images of two stroke patients using the proposed and existing methods. As shown, the proposed method achieved the most accurate denoising performance in lesion regions as indicated by the red arrows in the accompanied FLAIR images. (B) The distribution of CBF values within lesion and normal regions from different denoised results. TV, total variation.

To validate the clinical feasibility of the proposed method, we applied the proposed method to the denoising of ASL images from two stroke patients. Figure 5A summarizes the representative denoised CBF results with lesions using different methods. As can be seen, the proposed method achieved the best denoising performance with ischemic regions clearly delineated. On the contrary, existing denoising methods showed suboptimal denoising performance and limited lesion detectability. In Figure 5B, we further analyzed the distributions of the denoised CBF values in lesion and normal regions. As shown, the intensity distributions from normal and lesion regions were effectively separated after denoising, demonstrating the effectiveness of the proposed method in practical use. Further quantification of the lesion parts was summarized in Table S2.

## 5 Discussion

We proposed a novel DL‐based method for denoising ASL data. The method overcomes the practical issues of limited training data and biased results for novel features (e.g., lesions) that are not included in the training data. This was enabled by several key technical innovations. First, the proposed method mitigates limited‐data issues by leveraging the large public data via DSB‐based distribution remapping. This strategy integrates the populational variations in public data and the signal characteristics in limited local data to generate diverse synthesized data for model training, producing large training data to achieve good DL‐based denoising performance as shown in Figure 2. Second, rather than directly using the DL output as the final result, we use it only as a prior and explicitly recover the novel image components. This strategy effectively reduces the potential bias introduced by the DL model as shown in Figure 5.

The proposed method is effective in capturing both in‐ and out‐of‐distribution features; however, several aspects of the sparse modeling can be further improved. First, our current implementation uses a fixed sparsity constraint for novel feature recovery. Future work could explore adaptive or spatially varying sparsity constraint, in which the weighting of pixel‐wise sparsity depends on tissue class, anatomical region, or uncertainty estimated from the deep prior. Second, rather than pre‐assuming a specific sparsity form (e.g., TV‐based regularization), the sparsity constraint itself could be learned from data. For example, deep unrolled networks could be used to parameterize and learn the regularization structure within the iterative reconstruction process [66], enabling more data‐adaptive modeling of subject‐specific novel features. Third, our current implementation selects the optimal regularization weight, $\lambda_{\mathrm{reg}}$, on a per‐subject basis based on L‐curve, which is time‐consuming. Our previous work has shown that, for a fixed constrained reconstruction framework and a given class of image functions with comparable noise level, the optimal regularization parameter exhibits only weak inter‐subject variability [67]. Therefore, in future work, once sufficient training data become available, we plan to pre‐select a fixed global hyperparameter for application to new data and evaluate its generalizability. Finally, further enriching the training dataset to capture a broader physiological variability, including mild pathological cases, could strengthen the learned prior, thereby simplifying the residual structure and facilitating more reliable recovery of novel features.

## 6 Conclusions

We proposed a novel DL‐based method for denoising ASL data. The method overcomes the practical issues of limited training data and biased results for novel features that are not included in the training data. Evaluations based on both simulated and in vivo experimental data acquired using diverse ASL sequences demonstrated that the proposed method achieved effective and generalizable denoising performance. The proposed method has great potential to enhance the practical utility of ASL perfusion imaging.

## Funding

The authors have nothing to report.

## Conflicts of Interest

Rong Guo is currently an employee of Siemens Medical Solutions USA, Inc.

## Supporting information


**Figure S1:** Representative results from different slices of the stroke patient. (A) The population‐prior‐driven component is mostly consisted of the “in‐distribution” normal‐appearing image features. (B) The negative residual component displayed after sign inversion for visualization, highlighting the out‐of‐distribution sparse lesion features. (C) The final denoised image obtained by combining the population‐prior‐driven component with the signed residual component. (D) The reference image acquired with full averages.
**Figure S2:** Network architecture of the deep denoiser used in our work. This network integrates the U‐shape convolutional layers for local feature extraction, recurrent residual connections for effective feature propagation with attention gate modules for global context awareness.
**Figure S3:** Illustration of cross‐dataset discrepancies. For intuitive visual comparison, all datasets were nonlinearly warped into the standard MNI space using ANTs to ensure spatial alignment. One representative subject from each dataset is shown to highlight inter‐dataset variations in contrast, noise characteristics, and anatomical appearance. (Data from SPICE, ASLtbx, ADNI 3 serve as three different limited target datasets, while data from QTAB and PTBP serve as large public datasets).
**Figure S4:** Distribution‐remapping results on three ASL datasets acquired with different ASL sequences. The source CBF image is from the QTAB dataset, which was acquired using pCASL‐2D‐EPI sequence. The target ASL datasets were acquired using PASL‐3D‐EPSI (dataset 1), pCASL‐3D‐GRASE (dataset 2), and PASL‐2D‐EPI (dataset 3) sequences, respectively. During the diffusion translation process, the original public CBF map (*t* = 1) is progressively transformed toward the signal characteristics of the local CBF data. The remapped CBF images (*t* = 0) exhibit intensity and contrast that closely match the targeted local CBF maps, indicating successful distribution remappings. The translation performed consistently across the three datasets, confirming its generalization capability to handle different ASL sequences.
**Figure S5:** Illustration of remapping performance for PASL‐based datasets and pCASL‐based datasets. (A) CBF maps from the pCASL‐based public datasets. (B, D) CBF maps from different subjects acquired with PASL and pCASL, respectively. (C, E) Remapped CBF maps transformed from the public datasets. (F) GM intensity distributions of the public CBF maps, remapped maps and targeted maps for pCASL‐based and PASL‐based datasets and their alignment metrics. GM, gray matter, CDF, cumulative distribution function, JSD, Jensen‐Shannon divergence.
**Figure S6:** Ablation study of different data augmentation methods. (A) Representative data generated from histogram matching, cycle‐GAN and DSB. (B) Denoised CBF maps with different deep priors that were trained from five augmented training datasets (generic geometric transformations, histogram‐matching–based intensity transformation, cycle‐GAN–based intensity transformation, noise simulations based on parametric Rician noise models and DSB‐based intensity transformation).
**Figure S7:** In vivo experiments evaluating the reproducibility of the proposed method. (A) Denoised CBF maps obtained using different denoising methods from two repeated noisy ASL measurements of the same subject. (B) Bland–Altman plots comparing the CBF estimates between the two repetitions for each method. The proposed method achieved the smallest estimation bias and standard deviation among all methods, demonstrating superior reproducibility and consistency across repeated acquisitions. TV, total variation.
**Figure S8:** Comprehensive in vivo denoising results from a single subject across five independent repetitions. To simulate limited‐average conditions, noisy data were formed using the first 25 averages (with a total of 30 averages available), where every 5 averages constituted one noisy dataset. Four different denoising methods were applied for comparison. The results demonstrate consistent denoising performance and reproducibility across repeated acquisitions, highlighting the robustness of the proposed method under varying noise realizations.
**Figure S9:** In vivo denoising results from multiple subjects from PASL‐based datasets and pCASL‐based datasets. (A) The results showing a consistent denoising performance between (upper) PASL and (lower) pCASL datasets. (B) the quantitative group‐wise analysis of (left) PASL‐based datasets and (right) pCASL‐based datasets.
**Table S1:** Summary of datasets used in this study. High‐SNR CBF maps from two large public datasets (QTAB and PTBP, acquired with full averaging) were used as source datasets. Three target datasets (including a subset of the public ADNI3 dataset) acquired using distinct ASL sequences (differing in labeling pulses, readout strategies, spatial resolutions, and post‐labeling delays) [7, 17], were employed to evaluate the generalizability of the proposed distribution‐remapping framework. For each target dataset, high‐SNR reference data were reconstructed using full averaging of all acquired control/label pairs, while low‐SNR data were generated using only 15%–20% of the total averages to simulate limited‐average acquisition conditions.
**Table S2:** More detailed quantifications of denoising results from the stroke patient.

## Data Availability

The code and data that support the findings of this study are available from the corresponding author, upon reasonable request.
