# Supplementary material for: Denoising of ASL Data Using Deep Learning Priors Generated From Distribution Remapping
Source: Magn Reson Med. 2026 Jun 10;96(4):1972–82. doi: 10.1002/mrm.70471 (PMC13421004; doi:10.1002/mrm.70471)
Supplement: Supplementary file 1 — Figure S1: Representative results from different slices of the stroke patient. (A) The population‐prior‐driven component is mostly consisted of the “in‐distribution” normal‐appearing image features. (B) The negative residual component displayed after sign inversion for visualization, highlighting the out‐of‐distribution sparse lesion features. (C) The final denoised image obtained by combining the population‐prior‐driven component with the signed residual component. (D) The reference image acquired with full averages. Figure S2: Network architecture of the deep denoiser used in our work. This network integrates the U‐shape convolutional layers for local feature extraction, recurrent residual connections for effective feature propagation with attention gate modules for global context awareness. Figure S3: Illustration of cross‐dataset discrepancies. For intuitive visual comparison, all datasets were nonlinearly warped into the standard MNI space using ANTs to ensure spatial alignment. One representative subject from each dataset is shown to highlight inter‐dataset variations in contrast, noise characteristics, and anatomical appearance. (Data from SPICE, ASLtbx, ADNI 3 serve as three different limited target datasets, while data from QTAB and PTBP serve as large public datasets). Figure S4: Distribution‐remapping results on three ASL datasets acquired with different ASL sequences. The source CBF image is from the QTAB dataset, which was acquired using pCASL‐2D‐EPI sequence. The target ASL datasets were acquired using PASL‐3D‐EPSI (dataset 1), pCASL‐3D‐GRASE (dataset 2), and PASL‐2D‐EPI (dataset 3) sequences, respectively. During the diffusion translation process, the original public CBF map (t = 1) is progressively transformed toward the signal characteristics of the local CBF data. The remapped CBF images (t = 0) exhibit intensity and contrast that closely match the targeted local CBF maps, indicating successful distribution remappings. The translation performed con [file MRM-96-1972-s001.docx]

**Appendix A: I^2^SB Brief Recap**

I^2^SB is a recently proposed special variant of Diffusion Schrödinger Bridges (DSB) that enables domain-to-domain translation through stochastic optimal transport [1]. Mathematically, with paired two-domain images $\left( f_{\mathrm{src}},f_{\mathrm{tar}} \right)\sim\left( \Psi_{\mathrm{src}},\Psi_{\mathrm{tar}} \right)$, I^2^SB essentially is to gradually add noise onto the source-domain images to distort the images and denoise them back to the target-domain ones, like what most of the diffusion models are doing. The diffusion process was given by [1]:

$$f_{t}\sim q\left( f_{t}|f_{\mathrm{src}},f_{\mathrm{tar}} \right)\mathcal{=N}\left( f_{t}; \mu_{t}\left( f_{\mathrm{src}},f_{\mathrm{tar}} \right), \Sigma_{t} \right), t=0,1,\ldots T-1$$

where $\sigma_{t}^{2}$ and $\bar{\sigma}_{t}^{2}$ are variances predefined by a hyperparameter $\beta_{t}$, and $T$ represents the forward diffusion step number. And I^2^SB is trained to minimize bridge denoising loss [1]:

where $\epsilon_{\theta}\left( f_{t},t \right)$ denotes the noise-predictor for guiding the forward/backward process. With learned noise-predictor $\epsilon_{\theta^{*}}\left( f_{t},t \right)$, for any testing data sampled from the public domain $f_{\mathrm{src}}\sim\Psi_{\mathrm{src}}$, a DDPM-based posterior sampling method was used to iteratively generate intermediate samples beginning from $f_{T}=f_{\mathrm{src}}$ [2]:

where $\mu_{\theta}\left( f_{t},t \right)=\frac{1}{\sqrt{\alpha_{t}}}\left( f_{t}-\frac{\beta_{t}}{\sqrt{1-\bar{\alpha}_{t}}}\epsilon_{\theta^{*}}\left( f_{t},t \right) \right)$ and $\alpha_{t}=1-\beta_{t}$, $\bar{\alpha}_{t}=\prod_{s=1}^{t} \alpha_{s}$. The remapped target image can be finally obtained as $\tilde{f}_{\mathrm{tar}}=f_{0}$, such that

**Appendix B: Network Implementation**

As is described in the main manuscript, all CBF maps were spatially normalized to the MNI standard space and intensity-scaled to the range [0, 1]. The diffeomorphic transformations from the corresponding T1-weighted images to the MNI template were estimated using ANTs [3] and subsequently applied to the associated CBF maps. The resulting images in MNI space were then zero-padded to a size of 256×256 to match the input dimensions required by both distribution-remapping networks and prior generating networks.

**I. Distribution-Remapping-Based Data Augmentation**

In our work, we use two I^2^SB models for target training data augmentation, including:

First, to learn the intensity distribution remapping function, the public high-SNR CBF datasets from QTAB and PTBP was used [4, 5], which included a total of 750 data from health subjects acquired with pCASL-2D-EPI sequence. The high-SNR local CBF datasets were collected from several healthy volunteers (e.g. 7) using a special PASL-3D-EPSI sequence [6, 7] with full number of control/label pairs (e.g. 30). Specifically, each public CBF map was nonlinearly registered to each local subject using a diffeomorphic deformation model, generating structurally aligned pairs with differing intensity. We constructed pseudo-paired training data and then trained the DSB model for image-to-image translation. Once the first intensity-remapping I^2^SB is properly trained, given each public CBF data $f_{\mathrm{pub}}$, the remapped CBF can be generated as:

Similarly, to learn the measurement noise remapping function, noise associated with the target CBF images was obtained as the difference between high-SNR CBF maps (full averages, denoted as $f_{\mathrm{tar}}^{\mathrm{full}}$, e.g. 30) and low-SNR CBF maps (single or a few averages, denoted as $f_{\mathrm{tar}}^{\mathrm{few}}$, e.g. 5), $\varepsilon_{\mathrm{tar}}={(f}_{\mathrm{tar}}^{\mathrm{few}}-f_{\mathrm{tar}}^{\mathrm{full}})\sim\Psi_{n, tar}$. Gaussian noise was generated by sampling on the same spatial support (mask) as the source noise, $\varepsilon_{\mathrm{Gauss}}=\Omega⨀\eta$, where $\Omega\mathbb{=l}\left[ f_{\mathrm{tar}}^{\mathrm{full}}>0 \right]$ denotes the brain mask and $\eta\sim\mathcal{N}\left( 0,1 \right)$ denotes the whole spatial normal Gaussian noise. Once the second noise-remapping I^2^SB is properly trained, given each remapped target CBF $\tilde{f}_{\mathrm{tar}}$ and the corresponding mask support $\tilde{\Omega}\mathbb{=l}\left[ \tilde{f}_{\mathrm{tar}}>0 \right]$, the paired target noise can be generated from the masked Gaussian noise $\tilde{\varepsilon}_{\mathrm{Gauss}}=\tilde{\Omega}⨀\eta$:

**II. Deep-Learning-Based Prior Generation**

In our work, a U-Net-shaped network was utilized to learn the nonlinear mapping from low-SNR CBF image to its high-SNR counterpart [8], given its strong performance in learning image priors across medical imaging applications [9–11]. Given pairs of noisy CBF inputs and high SNR targets, ($\tilde{f}_{n},\tilde{f}_{r}$), as the training data, the network could be trained using a mean-squared-error loss:

Once the CBF deep denoiser is properly trained, given any low-SNR CBF map $f_{n}$, the high-SNR counterpart can be generated by $f_{r}^{*}=\mathcal{D}_{\theta}\left( f_{n} \right)$ as reference image.

In our work, an attention-ResUnet network was used to absorb the advantages of various network architectures by incorporating U-shape convolutional layers for local feature extraction, residual connections for effective feature propagation, and attention gate modules for global context awareness. This design optimally balances computational efficiency with denoising effectiveness.

All neural networks, including the Schrödinger diffusion bridges and the U-Net-based deep denoiser, were implemented in PyTorch [12] and trained using the Adaptive Moment Estimation (ADAM) optimizer [13]. The training processes were conducted on a workstation equipped with a single NVIDIA A6000 GPU (Santa Clara, CA), requiring approximately eight hours for diffusion bridge training and eight hours for denoiser training.

**Appendix C: Proposed Denoising Procedure**

In brief, given pretrained deep prior model $\mathcal{D}_{\theta}\left( \cdot\right)$ that translate low-SNR CBF maps to its high-SNR counterparts for generating deep priors, a parameter-predetermined Buxton model $\mathcal{A}_{\beta}\left( \cdot\right)$, and a measured noisy ASL difference image $\Delta M_{n}$, our proposed denoising procedure is basically composed of the following detailed steps:

1. Low-SNR CBF map $f_{n}$ is estimated from the measured low-SNR ASL difference image $\Delta M_{n}$ via the Buxton model, assisted by the acquired proton-density image for spatial regularization.
2. A diffeomorphic transformation model $\mathcal{T}$ that warps the low-SNR CBF map into the standard MNI space is estimated based on ANTs [3], for satisfying the input requirement of the deep learning model.
3. High-SNR CBF image prior $f_{r}^{*}$ is obtained by translating the low-SNR CBF map $f_{n}^{'}$ to its high-SNR counterpart using the pretrained deep prior model (slice by slice):
4. ASL reference component $\Delta M_{r}^{*}$ is obtained by first warping the high-SNR CBF prior back to the original space via the inverse diffeomorphic transformation in step 2 and then transforming the aligned high-SNR CBF prior to high-SNR reference component via the inverse Buxton model.
5. ASL sparse component $\Delta M_{s}^{*}$ is estimated by solving the following Bayesian-based optimization problem to recover the subject-dependent novel features:

where $W$ represents the matrix form of the total variation operator. The noise variance $\sigma^{2}$ is estimated from background voxels in the noisy ASL data $\Delta M_{n}$, and the regularization weight $\lambda_{\mathrm{reg}}$ is determined using the L-curve method. The resulting estimated optimal values are $0.0063\pm0.0013$, $0.016\pm0.0025$, and $0.03\pm0.0099$ for the local datasets 1, 2, and 3, respectively.

1. The final denoised ASL difference image $\Delta M_{t}^{*}$ can be reconstructed by adding up the reference component in step 4 and the sparse component in step 5, thereby obtaining physiologically consistent CBF quantity $f^{*}$ via the parameter-predetermined Buxton model:

**Appendix D: Performance Evaluations**

**Compared Denoising Methods**

To evaluate the denoising performance of the proposed method, we compared it against several state-of-the-art denoising methods commonly applied in ASL and medical imaging. TV functional form [14] was derived from the accompanying anatomical MPRAGE images to provide the edge-preserving constraints, and this method can be mathematically formulated as the following optimization problem:

where $W$ is the weighting estimated from the anatomical reference image that imposes spatial constraint for denoising. From a Bayesian perspective, incorporating statistical prior has shown its powerful ability in many denoising tasks [7, 15, 16], which solves the following optimization problem:

where $P_{r}\left( f \right)\mathcal{=N}\left( \mu_{f},\sigma_{f}^{2} \right)$ represents the prior distribution (characterized by mean and variance) estimated from large public datasets and $\lambda_{n}$ is determined based on the noise level.

**Quantitative Analysis**

The performance of all methods was assessed using a set of established quantitative analyses. Relative mean squared error (RMSE) was used to measure voxel-wise reconstruction accuracy relative to ground truth references. It is given by

where $\hat{x_{i}}$ and $x_{i}$ denote the voxel values from denoised image and the reference clean image, and $N$ is the total number of pixels. Global fidelity was assessed using the peak signal-to-noise ratio (PSNR). PSNR measures the logarithmic ratio between the maximum possible signal value, and the mean squared error (MSE) between the denoised image and the reference clean image, defined as:

where $MSE=\frac{1}{N}\sum_{i=1}^{N} \left( \hat{x_{i}}-x_{i} \right)^{2}$. Here $L$ represents the maximum pixel intensity. Structural similarity index measure (SSIM) evaluates perceptual quality in terms of contrast and structural preservation between $\hat{x}$ and $x$. It is given by

where $\mu_{x}$ and $\mu_{\hat{x}}$ denote mean intensities, $\sigma_{x}^{2}$ and $\sigma_{\hat{x}}^{2}$ are variances, and $\sigma_{x\hat{x}}$ represents covariance. $C_{1}$ and $C_{2}$ are small constant to stabilize the computation. Lower RSME, as well as higher PSNR and SSIM, indicates better denoising performance, with SSIM being particularly sensitive to structural preservation. To ensure robustness, all methods were tested across multiple repetitions, subjects, and different sites.

**Table S1.** Summary of datasets used in this study. High-SNR CBF maps from two large public datasets (QTAB and PTBP, acquired with full averaging) were used as source datasets. Three target datasets (including a subset of the public ADNI3 dataset) acquired using distinct ASL sequences (differing in labeling pulses, readout strategies, spatial resolutions, and post-labeling delays) [7, 17], were employed to evaluate the generalizability of the proposed distribution-remapping framework. For each target dataset, high-SNR reference data were reconstructed using full averaging of all acquired control/label pairs, while low-SNR data were generated using only 15–20% of the total averages to simulate limited-average acquisition conditions.

| **Datasets** | **Dataset 1 (SPICE)** | **Dataset 2 (ASLtbx)** | **Dataset 3 (ADNI 3)** | **QTAB** | **PTBP** |
| --- | --- | --- | --- | --- | --- |
| **Scanner** | Siemens 3T Prisma | Siemens 3T Trio | Siemens 3T Prisma | Siemens 3T Prisma | Siemens 3T Trio |
| **Sequence** | PASL | pCASL | PASL | pCASL | pCASL |
| **Readout** | 3D-EPSI | 3D-GRASE | 2D-EPI | 2D-EPI | 2D-EPI |
| **# Total Subjects** | 10 | 10 | 18 | 586 | 168 |
| **# Training** | 7 | 6 | 15 | N.A. | N.A. |
| **# Testing** | 3 | 4 | 3 | N.A. | N.A. |
| **Matrix Size** | $116\times117\times36$ | $64\times64\times34$ | $64\times64\times24$ | $128\times128\times24$ | $64\times64\times24$ |
| **# C/L pairs** | 30 | 6 | 54 | 15 | 40 |
| **# Pairs Used as**  **High-SNR Data** | 30 | 6 | 54 | 15 | 40 |
| **# Pairs Used as**  **Low-SNR Data** | 5 | 1 | 10 | N.A. | N.A. |
| **Time Saving** | 83.3% | 83.3% | 81.5% | N.A. | N.A. |
| **PLD (second)** | 1.8 | 0.8/1/1.5/2.2/3 | 1.2 | 1.5 | 1.2 |
| **Category** | Target Data | Target Data | Target Data | Source Data | Source Data |

**Table S2.** More detailed quantifications of denoising results from the stroke patient.

| **Method** | **SNR**  **(before denoising)** | **SNR**  **(after denoising)** | **RMSE** | **CBF**  **inside lesion** | **Lesion-to-normal**  **CBF ratio (mean)** | **p-value** |
| --- | --- | --- | --- | --- | --- | --- |
| **TV** | 2.1 | 3.4 | 9.8 | $23.1\pm7.9$ | 0.66 | p=0.09 |
| **Statistical** | 2.1 | 3.7 | 8.6 | $24.8\pm8.2$ | 0.83 | p=0.11 |
| **Deep Priors** | 2.1 | 4.5 | 6.1 | $24.7\pm9.2$ | 0.85 | p=0.10 |
| **Proposed** | 2.1 | 5.8 | 4.3 | $17.1\pm4.1$ | 0.55 | p<0.001 |
| **Reference** | 2.1 | 5.9 | N.A. | $16.9\pm4.2$ | 0.55 | p<0.001 |


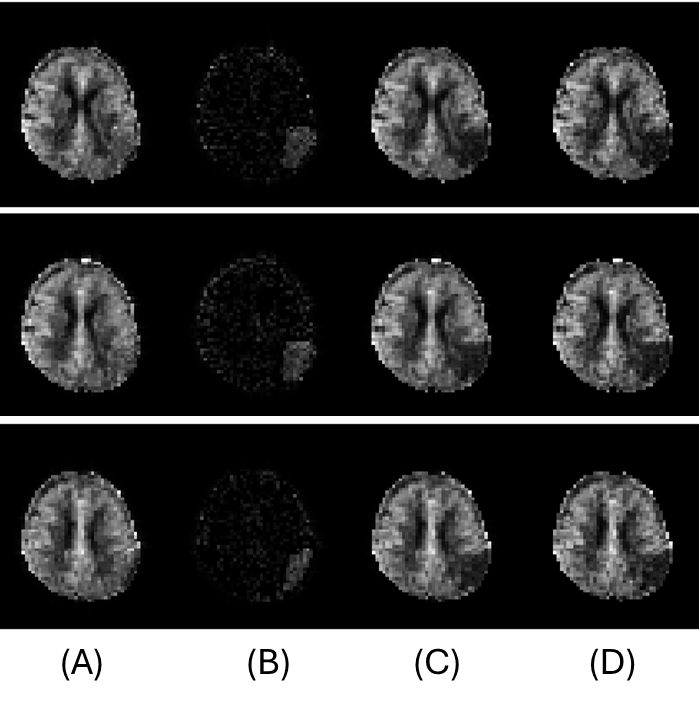


**Figure S1.** Representative results from different slices of the stroke patient. (A) The population-prior-driven component is mostly consisted of the “in-distribution” normal-appearing image features. (B) The negative residual component displayed after sign inversion for visualization, highlighting the out-of-distribution sparse lesion features. (C) The final denoised image obtained by combining the population-prior-driven component with the signed residual component. (D) The reference image acquired with full averages.


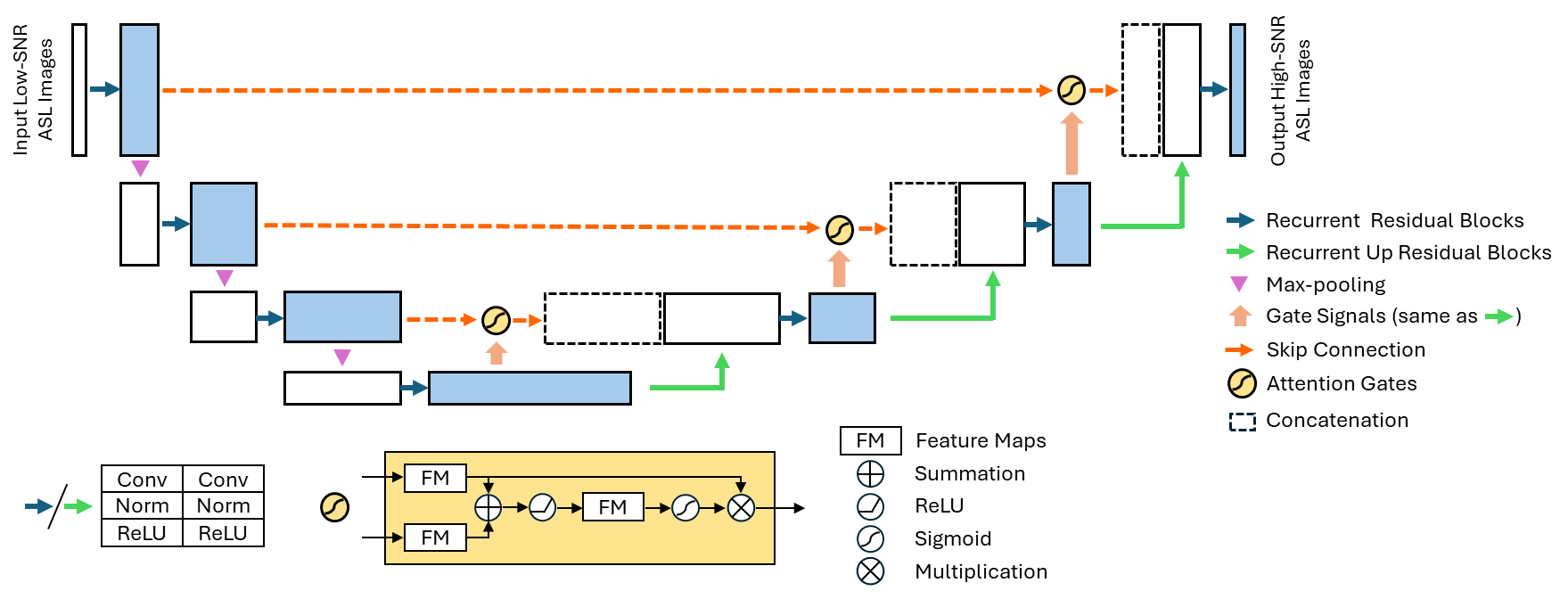


**Figure S2.** Network architecture of the deep denoiser used in our work. This network integrates the U-shape convolutional layers for local feature extraction, recurrent residual connections for effective feature propagation with attention gate modules for global context awareness.

**
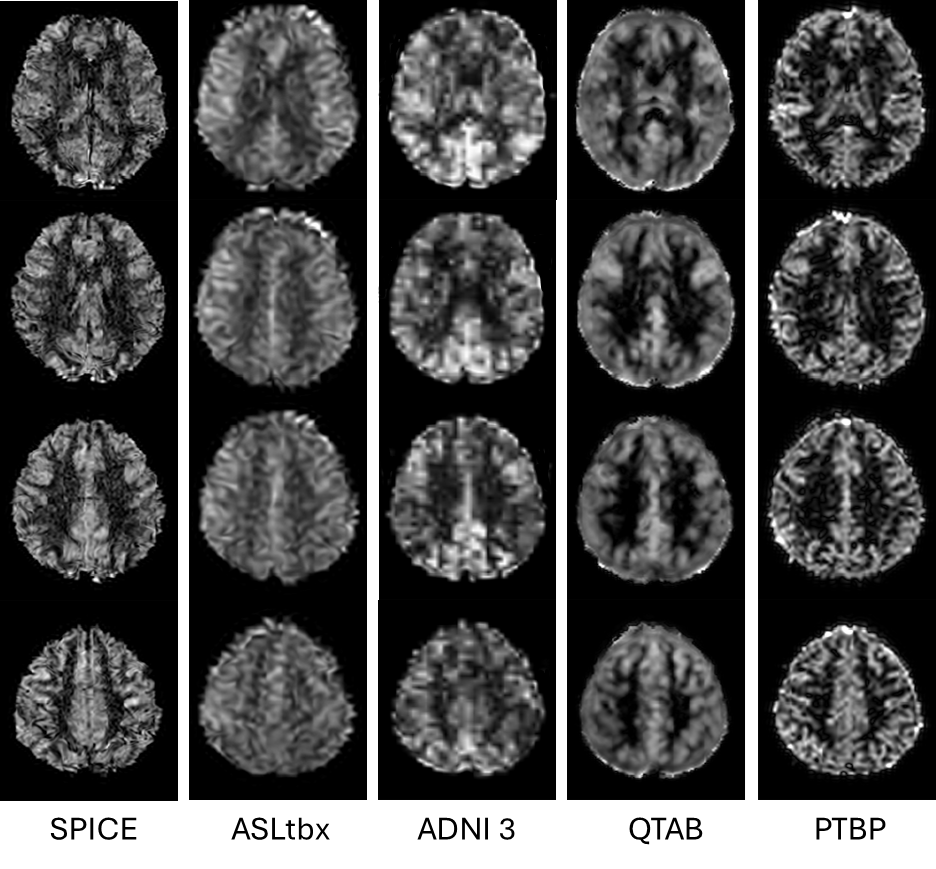
**

**Figure S3.** Illustration of cross-dataset discrepancies. For intuitive visual comparison, all datasets were nonlinearly warped into the standard MNI space using ANTs to ensure spatial alignment. One representative subject from each dataset is shown to highlight inter-dataset variations in contrast, noise characteristics, and anatomical appearance. (Data from SPICE, ASLtbx, ADNI 3 serve as three different limited target datasets, while data from QTAB and PTBP serve as large public datasets)

**
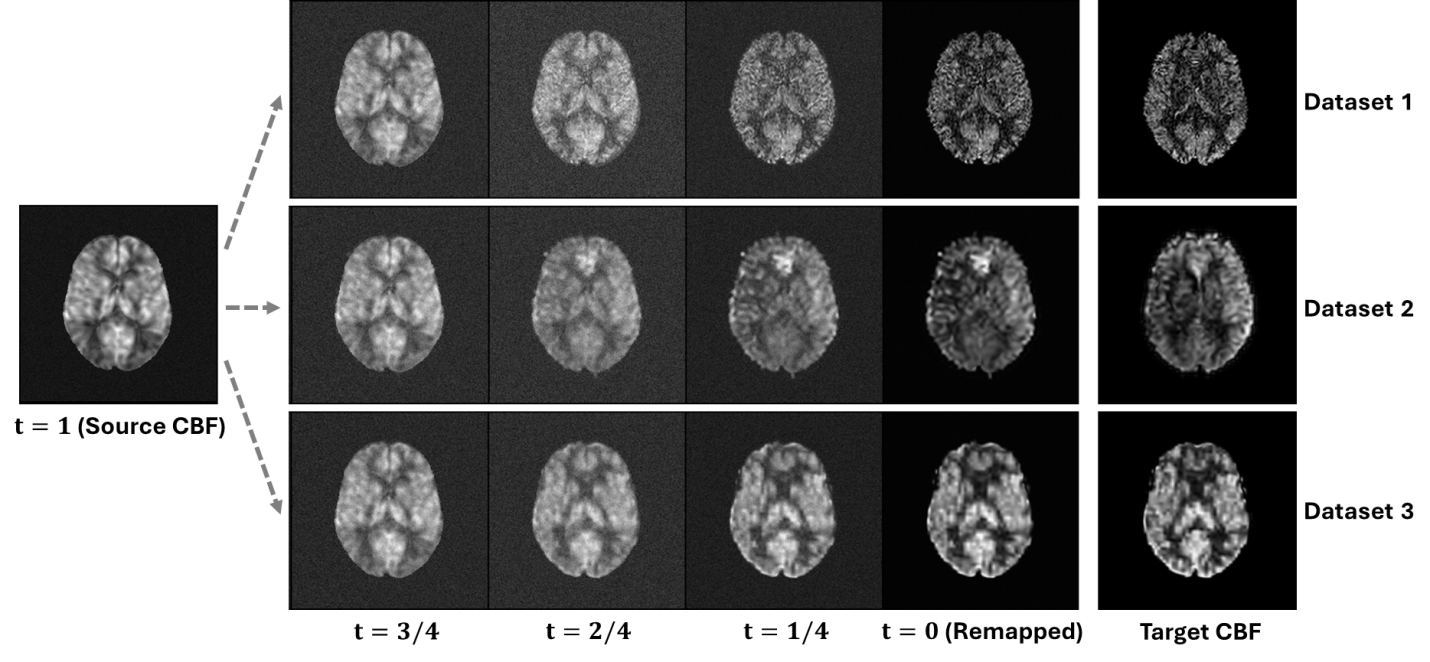
**

**Figure S4.** Distribution-remapping results on three ASL datasets acquired with different ASL sequences. The source CBF image is from the QTAB dataset, which was acquired using pCASL-2D-EPI sequence. The target ASL datasets were acquired using PASL-3D-EPSI (dataset 1), pCASL-3D-GRASE (dataset 2), and PASL-2D-EPI (dataset 3) sequences, respectively. During the diffusion translation process, the original public CBF map (t=1) is progressively transformed toward the signal characteristics of the local CBF data. The remapped CBF images (t=0) exhibit intensity and contrast that closely match the targeted local CBF maps, indicating successful distribution re-mapping. The translation performed consistently across the three datasets, confirming its generalization capability to handle different ASL sequences.

**
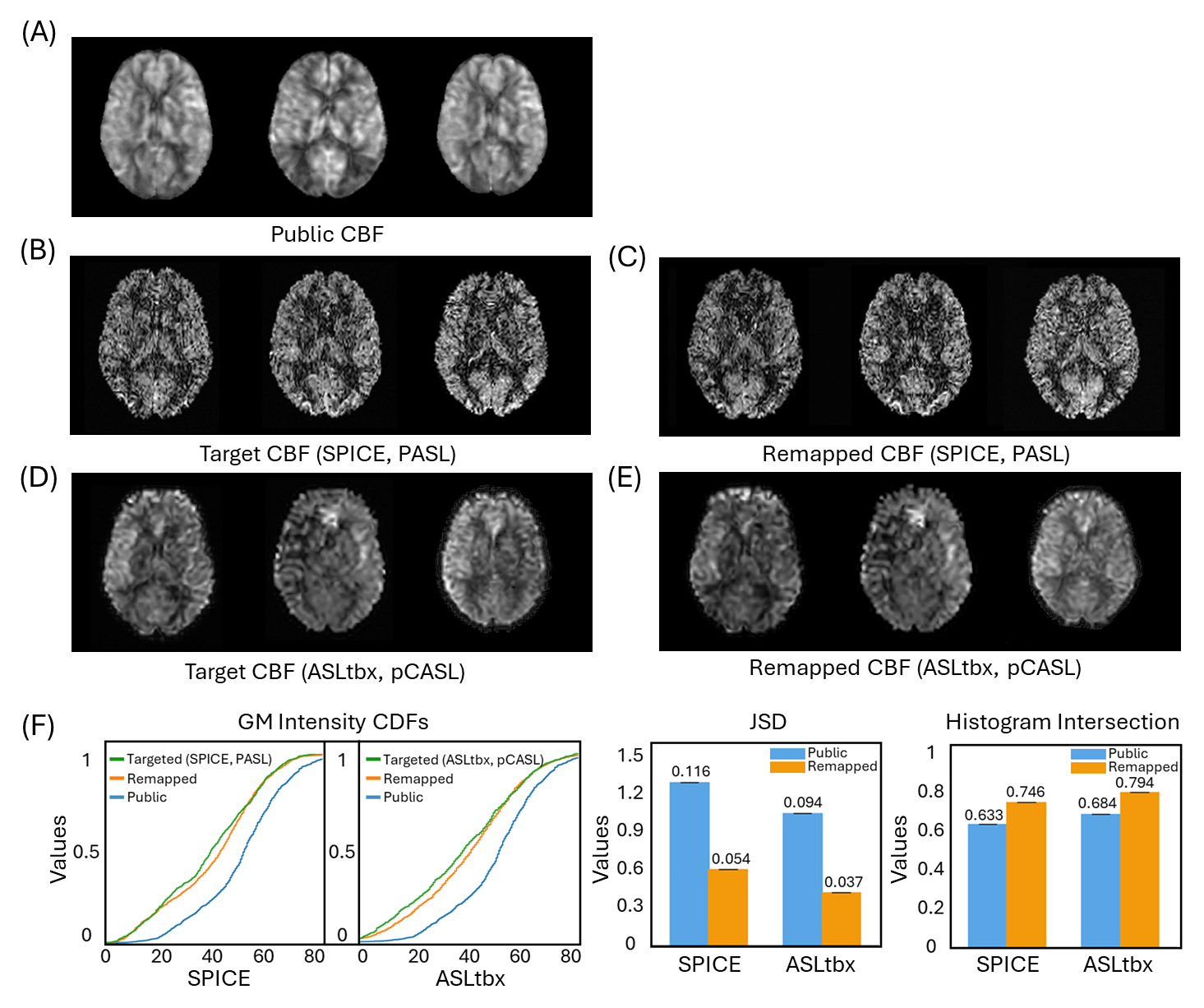
**

**Figure S5.** Illustration of remapping performance for PASL-based datasets and pCASL-based datasets. (A) CBF maps from the pCASL-based public datasets. (B)(D) CBF maps from different subjects acquired with PASL and pCASL, respectively. (C)(E) Remapped CBF maps transformed from the public datasets. (F) GM intensity distributions of the public CBF maps, remapped maps and targeted maps for pCASL-based and PASL-based datasets and their alignment metrics. Abbreviations: GM, grey matter, CDF, cumulative distribution function, JSD, Jensen-Shannon divergence.


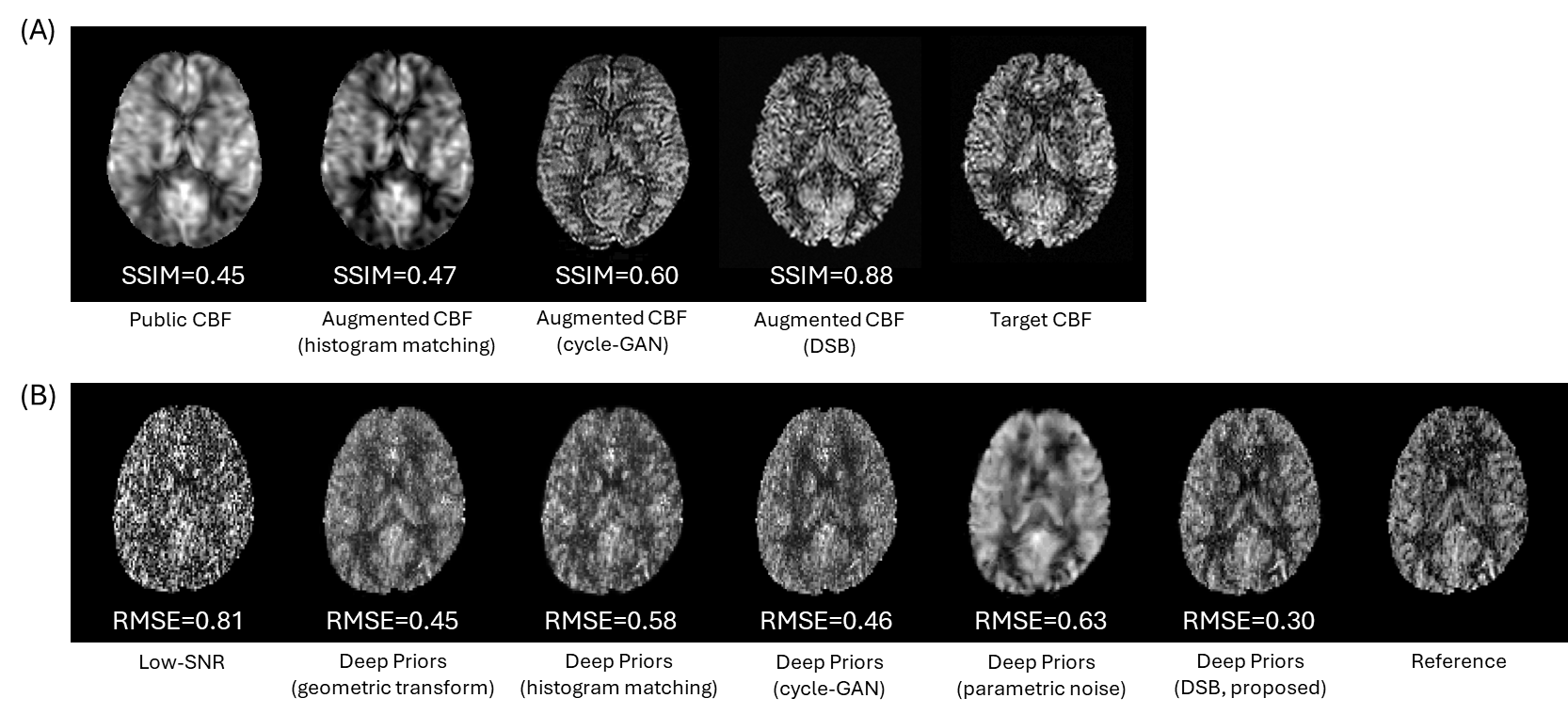


**Figure S6.** Ablation study of different data augmentation methods. (A) Representative data generated from histogram matching, cycle-GAN and DSB. (B) Denoised CBF maps with different deep priors that were trained from five augmented training datasets (generic geometric transformations, histogram-matching-based intensity transformation, cycle-GAN-based intensity transformation, noise simulations based on parametric Rician noise models and DSB-based intensity transformation.

**
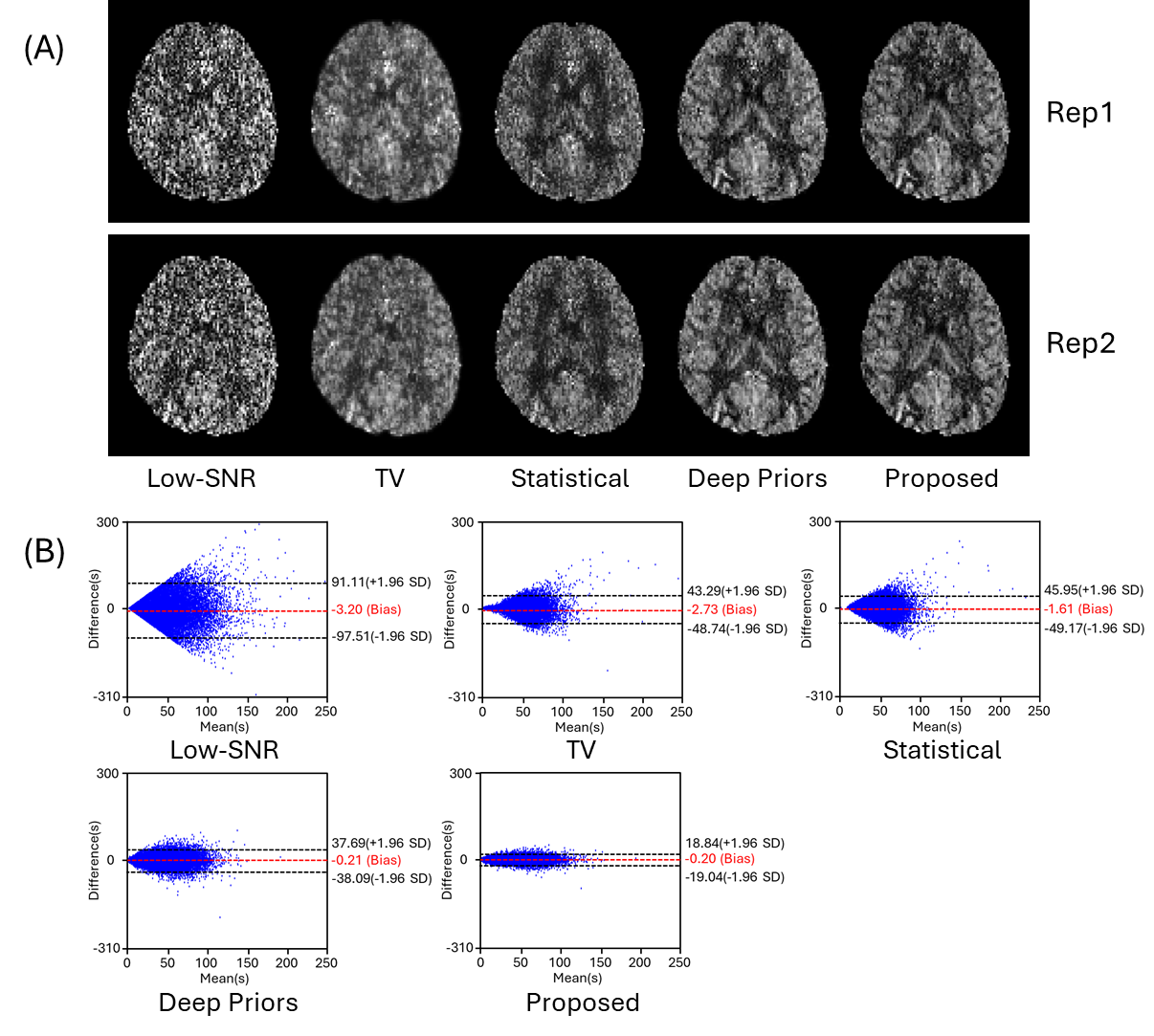
**

**Figure S7.** In vivo experiments evaluating the reproducibility of the proposed method. (A) Denoised CBF maps obtained using different denoising methods from two repeated noisy ASL measurements of the same subject. (B) Bland–Altman plots comparing the CBF estimates between the two repetitions for each method. The proposed method achieves the smallest estimation bias and standard deviation among all methods, demonstrating superior reproducibility and consistency across repeated acquisitions. Abbreviations: TV, total variation.


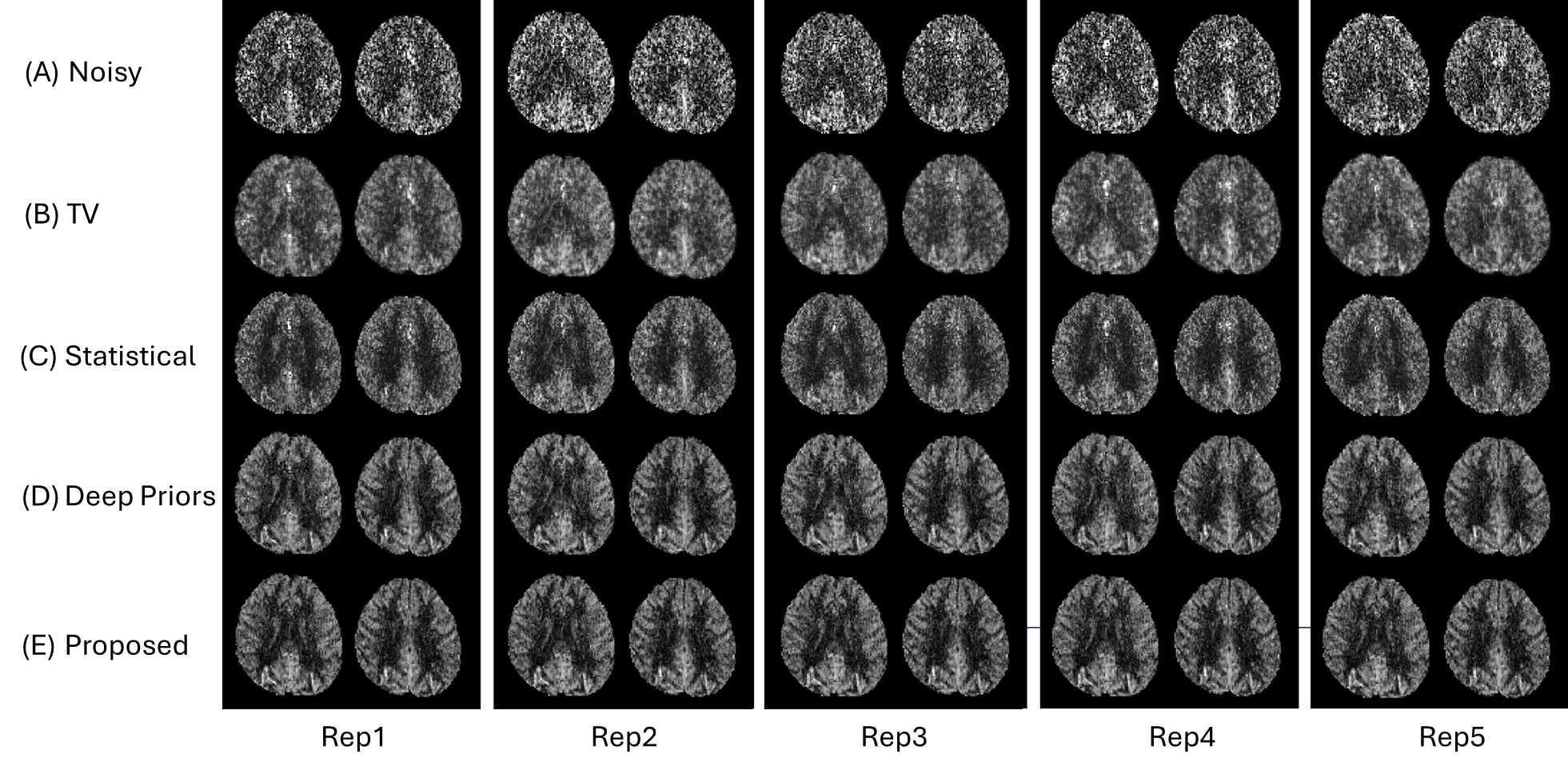


**Figure S8.** Comprehensive in vivo denoising results from a single subject across five independent repetitions. To simulate limited-average conditions, noisy data were formed using the first 25 averages (with a total of 30 averages available), where every 5 averages constituted one noisy dataset. Four different denoising methods were applied for comparison. The results demonstrate consistent denoising performance and reproducibility across repeated acquisitions, highlighting the robustness of the proposed method under varying noise realizations.

**
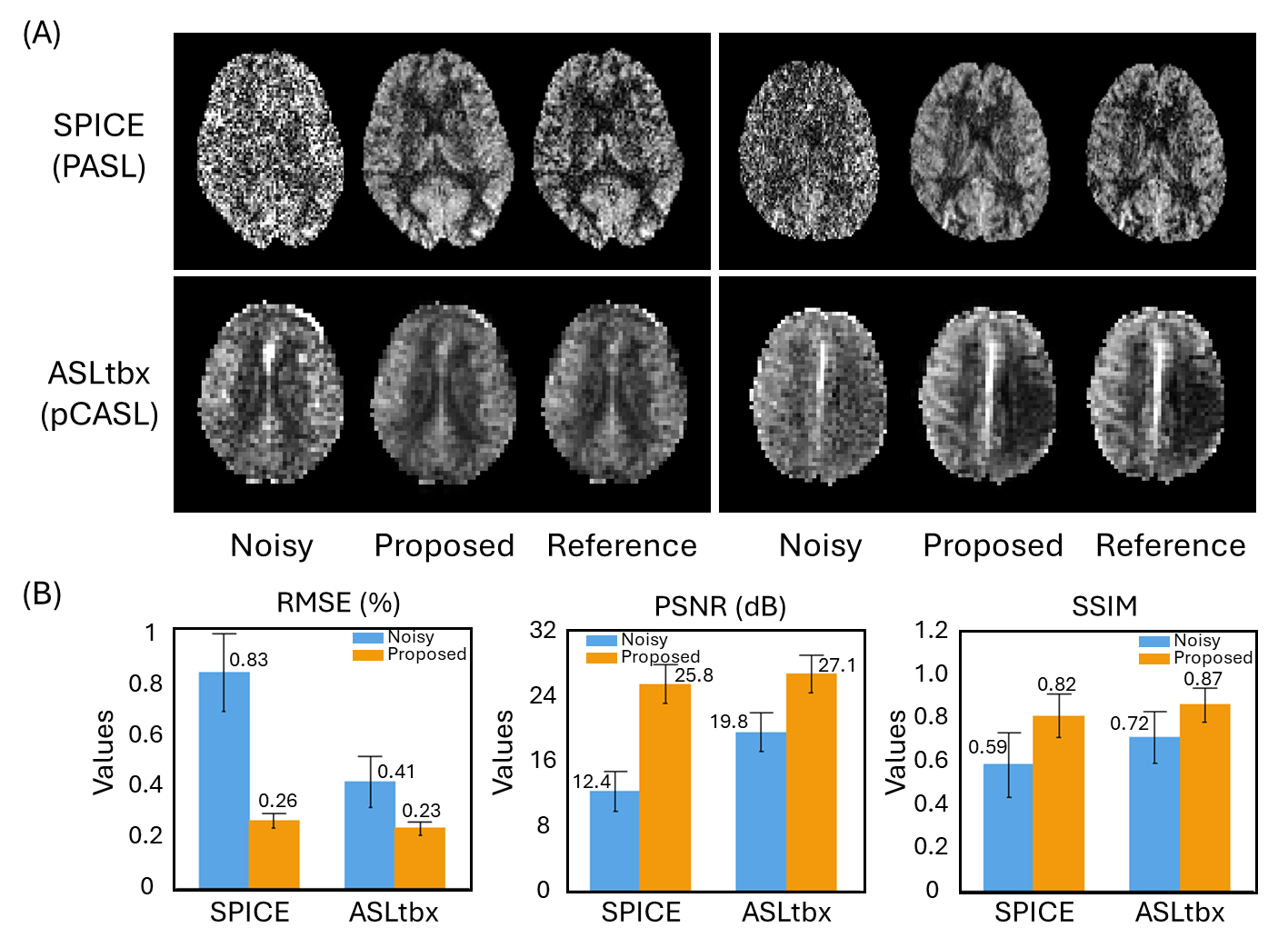
**

**Figure S9.** In vivo denoising results from multiple subjects from PASL-based datasets and pCASL-based datasets. (A) The results show a consistent denoising performance between (upper) PASL and (lower) pCASL datasets. (B) the quantitative group-wise analysis of (left) PASL-based datasets and (right) pCASL-based datasets.

**References**

1. Liu, G.-H., A. Vahdat, D.-A. Huang, et al., “I^2^SB: Image-to-Image Schrödinger Bridge,” (2023)

2. Ho, J., A. Jain, and P. Abbeel, “Denoising Diffusion Probabilistic Models,” (2020)

3. Avants, B. B., N. J. Tustison, G. Song, et al., “A Reproducible Evaluation of ANTs Similarity Metric Performance in Brain Image Registration,” *NeuroImage* 54, no. 3 (2011): 2033–2044. https://doi.org/10.1016/j.neuroimage.2010.09.025

4. Avants, B. B., J. T. Duda, E. Kilroy, et al., “The Pediatric Template of Brain Perfusion,” *Scientific Data* 2, no. 1 (2015): 150003. https://doi.org/10.1038/sdata.2015.3

5. Strike, L. T., N. K. Hansell, K.-H. Chuang, et al., “The Queensland Twin Adolescent Brain Project, a Longitudinal Study of Adolescent Brain Development,” *Scientific Data* 10, no. 1 (2023): 195. https://doi.org/10.1038/s41597-023-02038-w

6. Guo, R., X. Shao, Y. Li, et al., “High-Fidelity ASL Perfusion Imaging Using Unsuppressed Water Signals in MR Spectroscopic Imaging,” *Annual Meeting of International Society for Magnetic Resonance in Medicine* (2024): 1574

7. Guo, R., Z. Xu, Y. Li, et al., “Improved ASL in Water-Unsuppressed MRSI Using Generalized Series Modeling and Statistical Learning,” *Annual Meeting of International Society for Magnetic Resonance in Medicine* (2025): 3697

8. Lee JunHyun, “Implementation of U-Net, R2U-Net, Attention U-Net, Attention R2U-Net,” *https://github.com/LeeJunHyun/Image_Segmentation*

9. Ronneberger, O., P. Fischer, and T. Brox, “U-Net: Convolutional Networks for Biomedical Image Segmentation,” (2015)

10. Azad, R., E. K. Aghdam, A. Rauland, et al., “Medical Image Segmentation Review: The Success of U-Net,” *IEEE Transactions on Pattern Analysis and Machine Intelligence* 46, no. 12 (2024): 10076–10095. https://doi.org/10.1109/TPAMI.2024.3435571

11. Yuan, Y. and Y. Cheng, “Medical Image Segmentation with Unet-Based Multi-Scale Context Fusion,” *Scientific Reports* 14, no. 1 (2024): 15687. https://doi.org/10.1038/s41598-024-66585-x

12. Paszke, A., S. Gross, S. Chintala, et al., “Automatic Differentiation in PyTorch,” (2017)

13. Kingma, D. P. and J. Ba, “Adam: A Method for Stochastic Optimization,” (2017)

14. Rudin, L. I., S. Osher, and E. Fatemi, “Nonlinear Total Variation Based Noise Removal Algorithms,” *Physica D: Nonlinear Phenomena* 60, no. 1–4 (1992): 259–268. https://doi.org/10.1016/0167-2789(92)90242-F

15. Li, Y., Y. Zhao, R. Guo, et al., “Machine Learning-Enabled High-Resolution Dynamic Deuterium MR Spectroscopic Imaging,” *IEEE Transactions on Medical Imaging* 40, no. 12 (2021): 3879–3890. https://doi.org/10.1109/TMI.2021.3101149

16. Zhang, T., Y. Zhao, W. Jin, et al., “B1 Mapping Using Pre‐Learned Subspaces for Quantitative Brain Imaging,” *Magnetic Resonance in Medicine* 90, no. 5 (2023): 2089–2101. https://doi.org/10.1002/mrm.29764

17. Wang, Z., G. K. Aguirre, H. Rao, et al., “Empirical Optimization of ASL Data Analysis Using an ASL Data Processing Toolbox: ASLtbx,” *Magnetic Resonance Imaging* 26, no. 2 (2008): 261–269. https://doi.org/10.1016/j.mri.2007.07.003
